# Supplementary figures and images for: Extracellular Degradation Into Adenosine and the Activities of Adenosine Kinase and AMPK Mediate Extracellular NAD+-Produced Increases in the Adenylate Pool of BV2 Microglia Under Basal Conditions
Source: Front Cell Neurosci. 2018 Oct 18;12:343. doi: 10.3389/fncel.2018.00343 (PMC6200843; doi:10.3389/fncel.2018.00343)

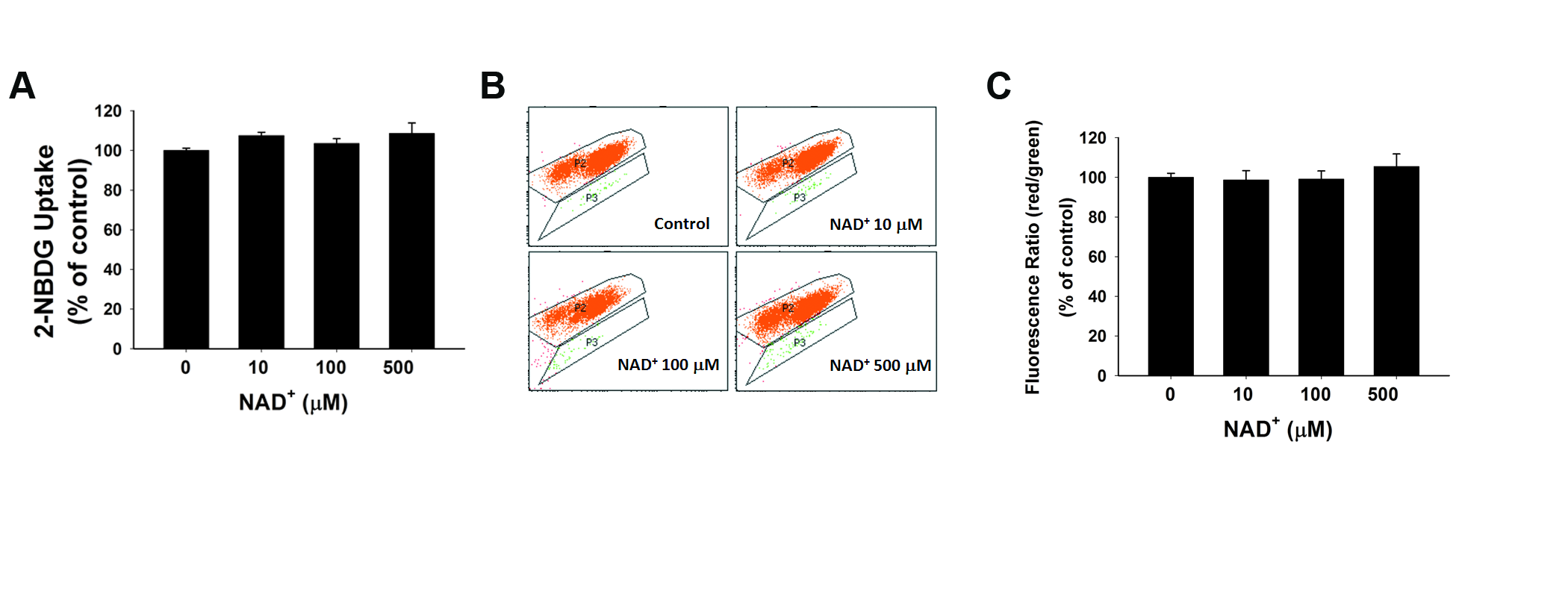

Supplement: FIGURE S1 — NAD+ treatment did not significantly affect the glycolytic rate or mitochondrial membrane potential of BV2 microglia under basal conditions. (A) 2-NBDG uptake assay did not show that the NAD+ treatment significantly affected the glycolytic rate of BV2 cells. (B) FACS-based JC-1 assay did not show that the NAD+ treatment affected the mitochondrial membrane potential of the cells. (C) Quantifications of the results from the FACS-based JC-1 assay did not show that the NAD+ treatment significantly affected the mitochondrial membrane potential of the cells. The cells were treated with NAD+ for 3 h. Subsequently, the assays were conducted. N = 12. The data were pooled from four independent experiments. [file Image_1.TIF]

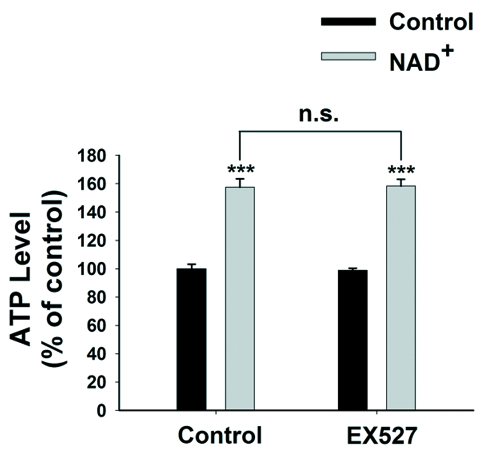

Supplement: FIGURE S2 — SIRT1 was not involved in the NAD+-induced increases of intracellular ATP levels. EX527, a selective SIRT1 inhibitor, did not affect the AMPK phosphorylation induced by NAD+ of BV2 microglia under basal condition. The cells were co-treated with 500 μM NAD+ and 10 μM EX527 for 3 h. Subsequently, Western blot assays were conducted. N = 12. The data were pooled from three independent experiments. ∗P < 0.05; ∗∗P < 0.01; ∗∗∗P < 0.001. [file Image_2.TIF]

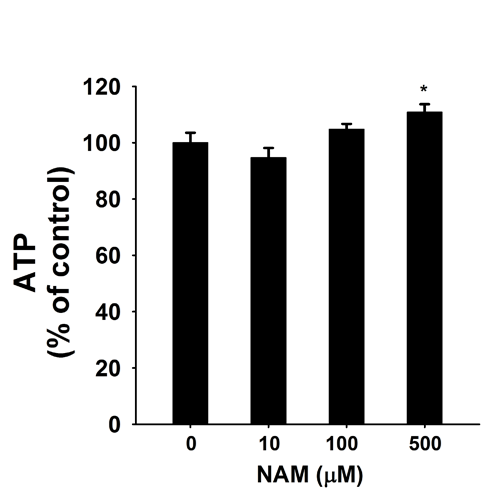

Supplement: FIGURE S3 — Nicotinamide treatment did not affect the intracellular ATP levels of BV2 microglia under basal condition. Treatment of the cells with 10 or 100 μM nicotinamide did not affect the intracellular ATP levels, while treatment of the cells with 500 μM nicotinamide slightly increased the intracellular ATP levels. The cells were treated with nicotinamide for 3 h. Subsequently, ATP assays were conducted. N = 12. The data were pooled from three independent experiments. ∗P < 0.05. [file Image_3.TIF]

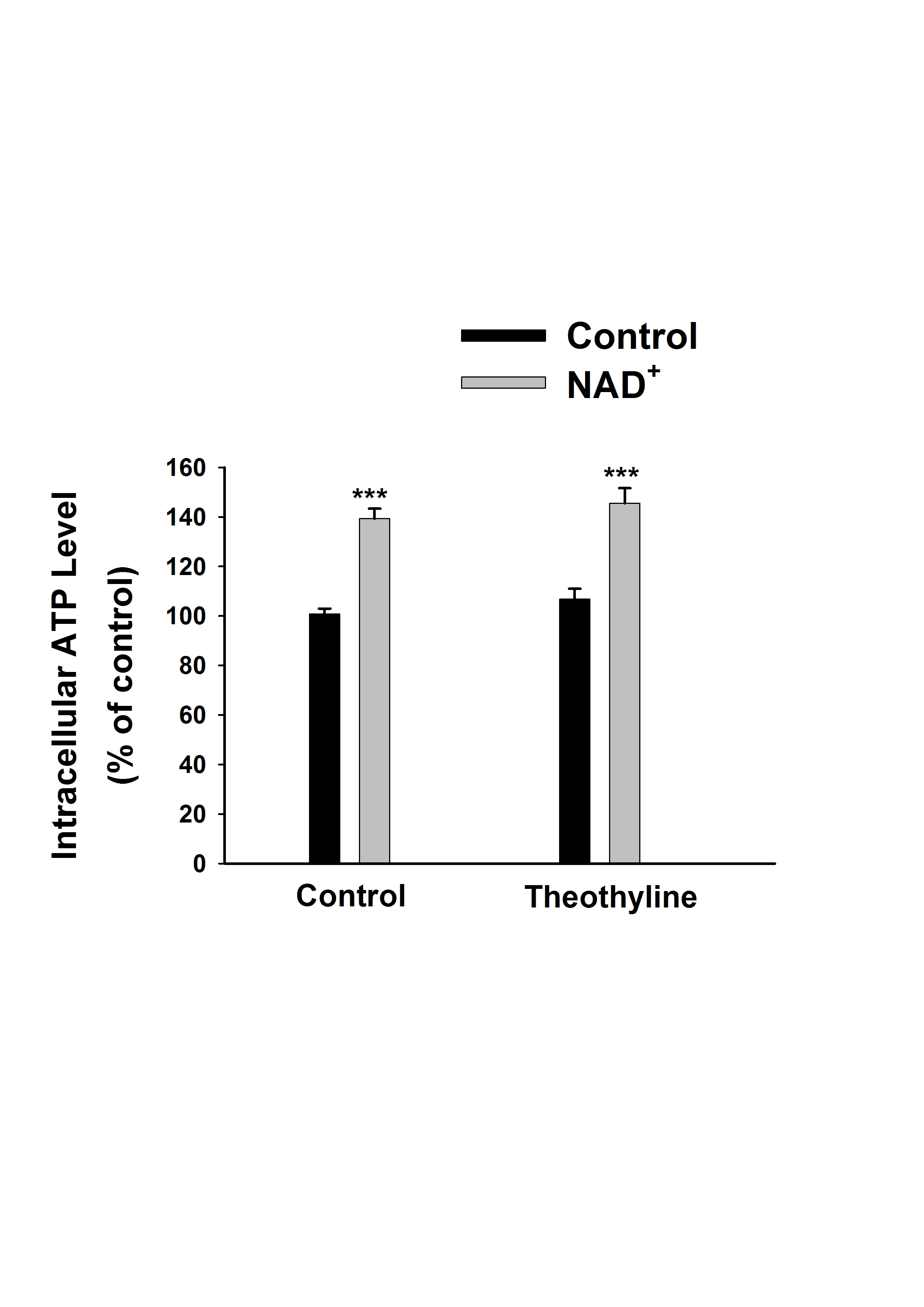

Supplement: FIGURE S4 — No implication of adenosine receptors in extracellular NAD+-induced increases in intracellular ATP level. Cells were co-treated with 1 μM and 0.5 mM NAD+ for 3 h. ∗∗∗P < 0.001. [file Image_4.TIF]

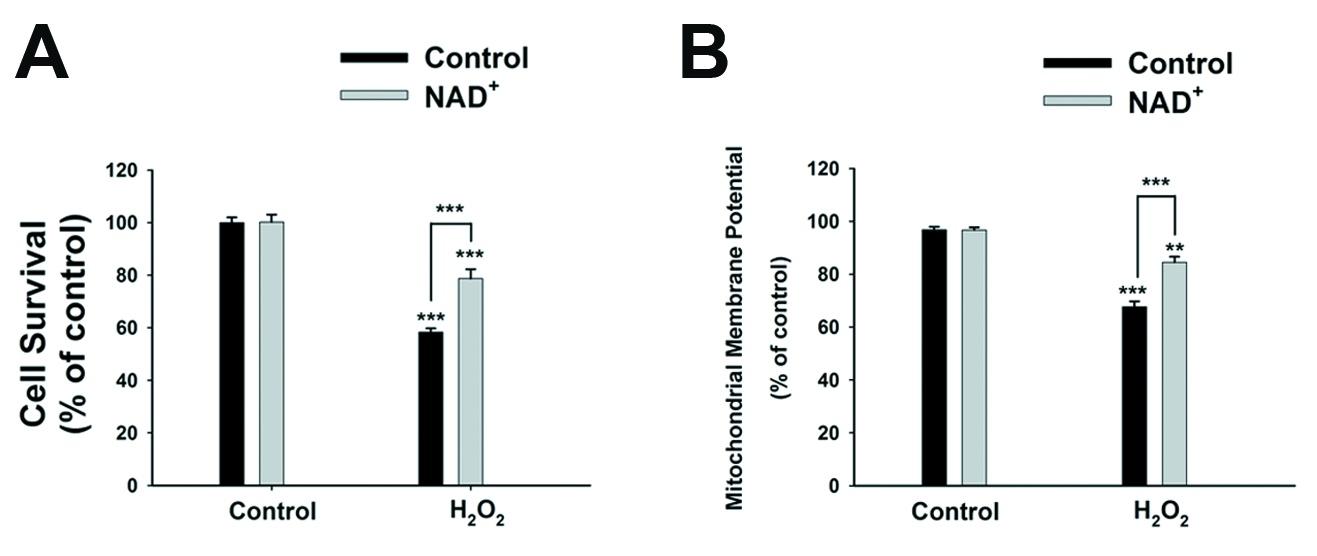

Supplement: FIGURE S5 — NAD+ treatment reduced hydrogen peroxide-induced cytotoxicity in BV2 cells. (A) Intracellular LDH assay showed that NAD+ treatment reduced H2O2 induced decrease in cell survival. (B) Flow cytometer based JC-1 assay showed that NAD+ treatment attenuated 1 mM H2O2 induced decrease in mitochondrial membrane potential. Cells were pretreated with 0.5 mM NAD+ for 3 h and then treated with 1 mM H2O2 for 1 h. ∗∗P < 0.01; ∗∗∗P < 0.001. [file Image_5.TIF]
